# The Role of Occupational Coping Self-Efficacy and Stressors in Intention-Related and Preparedness Outcomes among Ontario Baccalaureate Nursing Students

**DOI:** 10.1177/08445621261452362

**Published:** 2026-05-19

**Authors:** Madison Basso, Amanda McIntyre, Richard Booth, Ryan Chan

**Affiliations:** 1 Arthur Labatt Family School of Nursing, Western University, London, ON, Canada; 2 Department of Emergency Medicine, London Health Sciences Centre, London, ON, Canada

**Keywords:** students, nursing, self-efficacy, stress, career satisfaction, preparation, readiness

## Abstract

**Background:**

Nursing education is vital to addressing workforce shortages, with occupational coping self-efficacy potentially supporting student resilience, preparedness, and retention despite academic and clinical stressors. There is limited evidence examining how nursing students’ coping self-efficacy and perceived educational stressors relate to intention-related outcomes during training.

**Objective:**

To examine whether nursing students’ occupational coping self-efficacy in nursing (OCSE-N) and perceived stressors (Stressors in Nursing Students Scale; SNSS) differ across intention-related and preparedness variables including satisfaction with choosing nursing, intent to complete the Bachelor of Science in Nursing (BScN), and pursue further education, perceived preparedness for the registered nursing (RN) role, and preferred future work setting and location.

**Methods:**

A cross-sectional observational study was conducted using an online survey completed by 367 undergraduate nursing students in Ontario. Participants completed standardized measures of occupational coping self-efficacy (OCSE-N) and perceived stressors (SNSS), along with author-developed single-item measures assessing intention-related and preparedness indicators. OCSE-N and SNSS total scores were compared across groups using one-way analysis of variance with Bonferroni-adjusted post-hoc tests.

**Results:**

OCSE-N and SNSS scores differed significantly by satisfaction with choosing nursing and perceived preparedness for the registered nurse role, with higher OCSE-N and lower SNSS observed among students reporting greater satisfaction and preparedness. No significant differences were found across intent to practice as a nurse, plans to pursue further education, preferred work setting, or preferred work location. OCSE-N OCSE-N

**Conclusion:**

Occupational coping self-efficacy and perceived stressors were associated with satisfaction and perceived preparedness, but not with longer-term professional plans among Ontario baccalaureate nursing students.

## Background & Purpose

Recent data confirms the ongoing growing crisis in Canada's nursing workforce. In the most populous Canadian province of Ontario, nurse staffing ratios have consistently increased, with more than 25,000 additional nurses needed to meet the national average (Ontario Nurses Association, 2024). Despite ongoing efforts to increase the nursing supply, the province is falling further behind each year, with a projected shortfall of 33,000 nurses by 2027–2028 (Financial Accountability Office of Ontario, 2023). Several factors contribute to this deepening shortage, including the aging population, nursing burnout, young female-dominated profession with family responsibilities, population growth, and violence in the health setting (Haddad et al., 2023). Furthermore, internationally educated nurses encounter considerable barriers during the onboarding process, including challenges related to credential recognition, bridging requirements, workplace integration, communication norms, and organizational support, which impede their integration into the workforce (Rajpoot et al., 2024). These issues are compounded by provincial budget constraints that restrict adequate compensation for registered nurses, thereby hindering recruitment efforts, reducing job satisfaction, and contributing to high turnover rates (Registered Nurses’ Association of Ontario, 2021). High turnover rates and unfilled vacancies further exacerbate recruitment and retention efforts, creating a perpetual cycle of strain on Ontario's healthcare system (Ontario Nurses’ Association, 2022).

Nursing education is undeniably central to developing a sustainable nursing workforce. It is through nursing programs that students are prepared for the rigors of clinical practice and the complexities of providing quality patient care. It is important to note that baccalaureate nursing programs are uniquely positioned as the entry-to-practice pathway in Ontario, where undergraduate students face intensive academic, clinical, and licensing demands that shape their transition into the workforce (McMillan et al., 2023). Nursing education is not without its own significant challenges; faculty shortages, insufficient infrastructure, and the lingering effects of the COVID-19 pandemic impact enrollment in nursing programs and licensing rates for graduates (Buchan & Catton, 2023; Gazza, 2019; Hill, 2020). These structural challenges are also experienced directly by undergraduate students, who often face heavy workloads, reduced access to faculty mentors, and fewer clinical opportunities. Such conditions create academic and clinical stressors that directly affect students’ well-being and preparedness (Mohamed et al., 2024). These barriers hinder the growth of the future workforce, further amplifying the shortage.

While it is well-established that occupational stressors encountered during nursing education (e.g., clinical pressure, academic demands, and personal struggles) can significantly impact students’ well-being, (Günay & Kılınç, 2018; Ulenaers et al., 2021) less is known about how stressors, in combination with occupational coping self-efficacy, relate to intention-relevant outcomes during training. Occupational coping self-efficacy refers to an individual's confidence in their ability to cope with work-related demands and stressors (Laschinger et al., 2015). Higher occupational coping self-efficacy may support nursing students’ ability to manage educational and clinical demands and influence how prepared and satisfied they feel during training (Willie et al., 2025). Additionally, other coping resources (e.g., support from family, friends and institutions) play an important role in buffering students against occupational stress (Lavoie-Tremblay et al., 2022; Loureiro et al., 2024). Self-efficacy enables individuals to interpret challenges as opportunities for growth.

Given the critical importance of nursing education in addressing Ontario's nursing shortage, exploring the relationship between occupational coping self-efficacy, perceived stressors, and nursing students’ professional intentions is of paramount importance. For the purposes of this study, professional intentions are conceptualized as intention-related and preparedness-related indicators measured during training, including satisfaction with choosing nursing, perceived preparedness for the registered nurse role, intent to complete the Bachelor of Science in Nursing (BScN) and pursue further education, and preferred future work setting and location. These indicators reflect both proximal attitudes (e.g., satisfaction and preparedness) and longer-term preferences (e.g., practice setting and location), which may not be expected to relate to coping self-efficacy and stress in identical ways.

To address this gap, this study asks: (1) How do occupational coping self-efficacy scores vary across satisfaction with choosing nursing, intent to complete BScN and pursue further education, perceived preparedness for the registered nursing (RN) role, and preferred future work setting and location among Ontario baccalaureate nursing students? and (2) How do perceived educational stressors relate to these same intention-related variables?

Accordingly, our study aimed to examine nursing students’ occupational coping self-efficacy and perceived educational stressors during nursing and to compare these outcomes across professional intention and preparedness variables including satisfaction with choosing nursing, intent to complete the BScN and pursue further education, perceived preparedness for the RN role, and preferred future work setting and location.

## Methods & Procedures

### Design

This study employed a cross-sectional, non-experimental, observational design. Data were collected at a single time point using an online survey to capture sociodemographic and educational characteristics, intention-related and preparedness-related indicators, occupational coping self-efficacy, and perceived educational stressors among undergraduate nursing students in Ontario. The study adhered to the Strengthening the Reporting of Observational Studies in Epidemiology (STROBE) guidelines for cross-sectional studies. A completed STROBE checklist is available from the principal investigator upon request.

### Ethics and Consent

Ethics was approved from the Health Sciences Research Ethics Board at Western University (Project ID 123358). Informed consent was obtained prior to participation. Participants received a Letter of Information outlining the study's purpose, procedures, potential risks, benefits, and their rights. They were informed of their right to refuse or withdraw without penalty. Consent was documented through electronic acknowledgment before access. The voluntary nature of participation was emphasized. Confidentiality was maintained by collecting surveys anonymously and storing data securely with restricted access. The primary investigator's contact information was provided for questions or concerns. Finally, participants were offered the option to consent to receiving study results.

### Sample and Setting

Participants enrolled in a BScN program in Ontario, Canada were recruited to participate in an online survey from January to June 2024. At the time of recruitment, the College of Nurses of Ontario publicly listed 33 Ontario institutions offering Council-approved baccalaureate nursing (RN) programs (standalone or collaborative), and recruitment emails were distributed to all eligible institutions identified from this list. Programs eligible for a BScN in Ontario including the collaborative/direct entry and the compressed time frame program which includes the Registered Practical Nurse (RPN) pathway or bridging program. The collaborative/direct entry BScN program is designed for students applying directly from secondary school, or mature applicants. The compressed time frame BScN is an accelerated program that prepares students with previous post-secondary education (e.g., undergraduate degree), including those with training as an RPN (e.g., college diploma). Participants meeting the following inclusion criteria were eligible for participation: (a) aged 18 years or older; (b) enrolled in a baccalaureate nursing (BScN) program at an Ontario university (i.e., collaborative/direct entry, compressed timeframe, and RN bridging programs); and (c) could read and write in English. A formal a priori sample size calculation was not conducted due to the exploratory nature of this study. However, in 2019–2020 15,873 students were enrolled in a BScN program in Ontario (Canadian Association of Schools of Nursing, 2023).

### Recruitment

Participants were recruited from January to June 2024 using two strategies. First, using publicly available contact information, an email was sent to the director of nursing at all Ontario post-secondary education institutions with an eligible nursing program. The nursing student population was invited to participate in the study via an email sent from the program director. Reminder emails were distributed to maximize participation. Second, all professional nursing groups with an email list for general membership were contacted and invited to participate via email sent by executive members. Professional nursing groups included the Registered Nurses’ Association of Ontario Nursing Research Interest Group, Canadian Association of Schools of Nursing, and Canadian Nursing Students Association, as well as the nursing student association at each respective college or university. Professional organizations were included in the recruitment strategy because students often experience email fatigue from their educational institutions. To increase visibility of the study and bolster recruitment efforts, professional organizations who maintain a strong social media presence and wide membership reach were engaged. It was anticipated that this method would support greater recruitment success, as students frequently access and respond to opportunities shared through social media.

### Data Collection and Instrumentation

Participants who expressed interest after receiving the initial invitation email followed a link to a Qualtrics survey. After reviewing the letter of information and providing consent, they completed the survey directly on the platform.

To provide a comprehensive description of the sample, data were collected on sociodemographic variables (e.g., age, sex, gender, primary language spoken at home, cultural background, marital status, housing, and living accommodations) socioeconomic variables (e.g., employment status, hours of employment, and location of employment). Participants also reported their nursing program characteristics (i.e., program type, number of clinical hours, and number of months completed).

The intention-related and preparedness-related indicators (grouping variables) were measured in alignment with the study aim: (1) satisfaction with choosing nursing; (2) intent to complete the BScN and pursue further education; (3) perceived preparedness for the RN role; and (4) preferred future work setting and location. Participants were asked two additional questions rated on a 5-point Likert scale: “Right now, do you feel prepared to manage future work as a Registered Nurse?” (scores ranged from ‘1’ extremely unprepared to ‘5’ extremely prepared) and “How satisfied are you right now with your choice in pursuing Registered Nursing as a career?” (scores ranged from ‘1’ extremely dissatisfied to ‘5’ extremely satisfied). These items were included to capture proximal preparedness and satisfaction during training (i.e., intention-relevant indicators), and were used as categorical grouping variables for comparisons of OCSE-N and Stressors in Nursing Students Scale (SNSS) total scores

The dependent variables, OCSE-N and stressors in nursing, were collected using two standardized questionnaires: Occupational Coping Self-Efficacy for Nurses (OCSE-N) (Pisanti et al., 2008), and SNSS (Deary et al., 2003).

#### Occupational Coping Self-Efficacy for Nurses (OCSE-N)

The OCSE-N was created by Pisanti et al. (2008) in Italy to assess nurses’ confidence in coping with occupational stressors. OCSE-N consists of nine items rated on a 5-point Likert scale from 1 equating to *not at all easy to cope with* to 5 equating to *totally easy to cope with*. A sample item is: “How easily can you cope with caring for patients who are uncooperative?”). Total scores range from 9 to 45, with higher scores indicating greater coping self-efficacy (Laschinger et al., 2015). In nursing populations, the scale has demonstrated adequate internal consistency (Cronbach's alpha ranging 0.77–0.79) (Pisanti et al., 2008) and scale reliability (Pisanti et al., 2015). The tool has also been used for studying student nurse samples in the Canadian context (Laschinger et al., 2018). In the present study, the scale demonstrated good internal consistency (Cronbach's α = 0.83).

#### Stressors in Nursing Students Scale (SNSS)

The SNSS was developed in Scotland by Deary et al. (2003) to measure sources of stress specific to nursing students. It includes 43 items rated on a 5-point Likert scale from ‘1’ (not at all stressful) to ‘5’ (extremely stressful). A sample item is: “*Pressure of academic work while on placement.”* Total scores range 43 to 215 with higher scores reflecting greater perceived stress. Deary et al. (2003) reported good internal consistency for the original scale (Cronbach's alpha = 0.82−.83). Subsequent studies have examined its psychometric properties, including a principal components analysis confirming internal consistency in a Hong Kong nursing student sample (Watson et al., 2010) and the development and validation of a shortened version demonstrating reliability and validity in student populations (Salamonson et al., 2011). In the present study, the scale demonstrated excellent internal consistency (Cronbach's α = 0.94).

The OCSE-N and SNSS instruments were imported into Qualtrics with their original item wording and response options, using the same 5-point scoring anchors specified by the scale developers. Responses were recorded in Qualtrics and total scores were computed in SPSS according to published scoring procedures (OCSE-N total 9–45; SNSS total 43–215).

### Data Analysis

All electronic data were imported from Qualtrics into SPSS V25.0 (IBM Corp. 2018). Statistical significance was set at *p* < .05. Descriptive statistics were calculated for all demographic and study variables. Continuous variables were reported as means with standard deviations, and categorical variables as counts and percentages.

The dependent variables were OCSE-N and SNSS total scores. These outcomes were compared across the professional intention variables listed above (e.g., satisfaction with choosing nursing, preparedness for the RN role, intent to practice as an RN).

The independent variables were the categorical groups within each professional intention variable. One-way analysis of variance (ANOVA) with Bonferroni-adjusted post-hoc tests was conducted to determine whether mean OCSE-N or SNSS scores differed significantly between groups.

## Results

Of the 550 participants who initiated the survey, 367 completed all items on the OCSE-N and SNSS, yielding an attrition rate of 33.3%. For all participants who completed the survey, item-level missing data were minimal (<2% across demographic and educational variables, and none for OCSE-N or SNSS items). In contrast, participants who did not complete the survey demonstrated high levels of missingness across all variables, with all exceeding 50% missing. Consistent with the inclusion criteria, only participants with complete OCSE-N and SNSS data were included in the analyses (N = 367). No imputation was performed.

### Sociodemographic and Education Characteristics

Table 1 shows detailed sociodemographic and education characteristics for the study sample. Sociodemographic and educational characteristics are presented to describe the sample and contextualize findings; these variables were not examined as predictors of OCSE-N or SNSS. Participants were predominantly between 18 and 25 years of age (83.9%) and identified as female (91.8%) and as women (90.7%). Most participants reported English as their primary language spoken at home, and over half identified as being of European descent, with additional representation from East/Southeast Asian, African/Black, Indigenous, and other backgrounds.

**Table 1.** Sociodemographic and Education Characteristics for Total Sample (N = 367).

Characteristic	n	%
Age (years)		
18–25	308	83.9
26–39	48	13.1
40–59	5	1.4
Missing / prefer not to answer	6	1.6
Sex		
Female	337	91.8
Male	30	8.2
Gender identity		
Woman	333	90.7
Man	28	7.6
Non-binary / other	6	1.6
Primary language spoken at home		
English	309	84.2
Other	58	15.8
Cultural background / race		
European	204	55.6
East / Southeast Asian	45	12.3
African / Black	51	13.9
Indigenous	8	2.2
Other / unknown	59	16.1
Currently employed		
Yes	235	64.0
No	132	36.0
Pre-nursing healthcare experience		
Yes	110	30.0
No	257	70.0
Type of nursing program		
Direct-entry BScN (stand-alone or collaborative)	289	78.8
Second-entry compressed / accelerated	59	16.1
RPN bridge / pathway	15	4.1
Missing	4	1.1
Nursing studies completed (months)		
0–6	110	30.0
7–12	56	15.3
13–18	77	21.0
19–24	41	11.2
25–36	76	20.7
37–48	7	1.9
Clinical practice hours completed		
0	117	31.9
1–50	26	7.1
51–100	17	4.6
101–300	101	27.5
>300	105	28.6
Missing	1	0.3
First-generation student		
Yes	86	23.4
No	280	74.5
Missing	1	0.3

*Note*. Percentages may not total 100% due to rounding or missing responses. Sociodemographic and educational variables are presented for descriptive purposes only.

Most participants were enrolled in a direct-entry baccalaureate nursing program and were studying full-time. Approximately half of the sample had no prior post-secondary education, and nearly three-quarters were not first-generation students. Participants represented a range of progression through their nursing programs, with over two-thirds having completed some clinical practice hours.

Approximately two-thirds of participants reported being currently employed, and a minority reported prior healthcare-related work experience before entering nursing education.

### Professional Nursing Intentions and Perceived Clinical Ability

Most of the sample reported being satisfied with their choice of pursuing a career as an RN (*n* = 283, 77.1%). Of note, all participants planned to complete their BScN program, and all planned to obtain an RN licensure (i.e., pass the licensing exam) following graduation (*n* = 367, 100% for both). Although most of the sample planned to practice as an RN (*n* = 343, 93.5%), a smaller portion (*n* = 24, 6.5%) reported they were either uncertain or did not plan to practice as an RN following graduation. One third of the sample (*n* = 129, 35.1%) planned to pursue additional post-secondary education after graduation, and an additional 45.2% (*n* = 166) were uncertain. Therefore, 19.3% (*n* = 71) of participants did not intend to pursue additional post-secondary education after completion of the BScN.

A similar number of students reported being prepared (*n* = 146, 39.8%) and unprepared for the RN role (*n* = 140, 38.2%). Participants reported their preferred practice setting to be in acute care or hospitals (*n* = 255, 69.5%). Other practice areas of interest included public health (*n* = 32, 8.7%), government or professional institutions (*n* = 19, 5.2%), primary care (*n* = 18, 4.9%), or education (*n* = 13, 3.5%). Furthermore, most participants preferred to work locally (i.e., near their permanent residence) (*n* = 203, 55.3%). Other participants preferred to somewhere else within Ontario (*n* = 87, 23.7%), or outside of Ontario but within Canada (*n* = 35, 9.5%). Interestingly, 9.8% (*n* = 36) of the sample planned to pursue employment internationally.

### OCSE-N and SNSS

Mean scores for OCSE-N items ranged 2.6 to 3.2 which represents moderate OCSE. The lowest self-efficacy scores were reported on the items *dealing with insufficiently defined procedures* (*x* = 2.6, SD 0.9) and *physical tiredness* (*x* = 2.8, SD 1.1). Participants perceived having low-moderate coping ability for dealing with insufficiently defined procedures and physical tiredness.

Mean scores for SNSS items ranged 2.3 (low-moderate stress) to 3.8 (very high stress). Participants reported that the greatest source of stress was related to the *amount of classwork material to be learned* (x = 3.8, SD 1.0), followed by *fear of examinations and placement gradings* (x = 3.6, SD 1.1), *fear of making a mistake in clinical placements* (x = 3.6, SD 1.2), and *fear of failing in the course* (x = 3.6, SD 1.4). Participants reported that the least stressful items were related to *conflicts with peers* (x = 2.3, SD 1.1), *competition from fellow participants* (x = 2.3, SD 1.1), *relationships with family members* (x = 2.4, SD 1.2), and *conflicts with college staff* (x = 2.4, SD 1.1). Overall, participants perceived the greatest stress related to the amount of classwork, examinations and clinical evaluations, and fear of making mistakes in clinical placements or failing the course. Descriptive statistics for occupational coping self-efficacy and perceived educational stressors are presented below to contextualize subsequent comparisons across intention-related and preparedness indicators.

### Associations Between OCSE-N, SNSS, and Professional Intention Indicators

Table 2 compares OCSE-N and SNSS total scores by various professional nursing intention variables among the total sample. There were no significant differences in OCSE-N or SNSS total scores on the following variables: plan to practice as an RN post-graduation (*p* = 0.772 and *p* = 0.564, respectively), plan to pursue further post-secondary education (*p* = 0.751 and *p* = 0.393), preferred work setting (*p* = 0.243 and *p* = 0.375), and preferred work location (*p* = 0.732 and *p* = 0.621).

**Table 2.** Occupational Coping Self Efficacy in Nurses (OCSE-N) and Stressors in Nursing Students Scale (SNSS) Total Scores for Various Professional Nursing Intention Variables among the Total Sample (N = 367).

Variables	Sample		OCSE-N			SNSS		
	#	%	Mean	SD	p value	Mean	SD	p value
Total sample	367	100	27.8	5.36		129.1	26.9	
Satisfaction with Choice to Pursue Nursing^a^					.004			<.001
Dissatisfied (scores 1 or 2)	39	10.6	26.4	5.8		143.8	24.7	
Neutral (score of 3)	45	12.3	25.8	5.3		141.7	23.6	
Satisfied (scores 4 or 5)	283	77.1	28.4	5.6		125.1	26.5	
Plan to practice as RN					.772			.564
Yes	343	93.5	27.8	5.5		128.9	27.1	
Uncertain or no	24	6.5	28.2	6.8		132.2	24.6	
Plan to pursue further education					.751			.393
Yes	129	35.1	27.7	5.9		128.5	28.3	
Prefer not to answer/ Uncertain	167	45.5	27.8	5.3		130.9	25.6	
No	71	19.3	28.3	5.9		125.8	27.3	
Preparedness for RN role^b^					<.001			<.001
Disagree (scores 1 or 2)	140	38.1	25.4	5.4		137.0	24.7	
Neutral (score of 3)	81	22.1	27.5	4.6		131.6	24.3	
Agree (scores 4 or 5)	146	39.8	30.4	5.2		120.1	27.8	
Preferred work setting					.243			.375
Hospital or Acute Care	255	69.5	28.3	5.1		127.3	26.3	
Primary or Community Care	34	9.3	26.4	6.4		132.9	29.5	
Public Health	32	8.7	27.0	6.1		131.0	25.7	
Education or Government	32	8.7	26.9	7.5		135.5	29.7	
Other/Prefer not to answer	14	3.8	27.7	6.2		133.9	27.0	
Preferred work location					.732			.621
Local (in or around their permanent residence)	203	55.3%	27.9	5.2		127.5	26.4	
Provincial (another city within Ontario)	87	23.7%	27.3	6.1		130.5	25.1	
National (outside of Ontario, within Canada)	35	9.5%	28.7	5.5		130.2	27.3	
International (outside of Canada)	36	9.8%	27.9	7.1		131.4	32.7	
Prefer not to answer	6	1.6%	29.0	4.5		142.0	31.7	

^a^Assessed on a 5-point Likert scale ranging from extremely dissatisfied (‘1’) to extremely satisfied (‘5’).

^b^Assessed on a 5-point Likert scale ranging from extremely unprepared (‘1’) to extremely prepared (‘5’).

*Note*. OCSE-N = occupational coping self efficacy in nurses tool; SNSS: stressors in nursing students scale.

OCSE-N and SNSS scores were statistically different among participants with respect to their satisfaction to pursue nursing (*p* = 0.004 and *p* < 0.001). Participants who were satisfied with their choice to pursue nursing had significantly higher OCSE-N (*p* < 0.001) and lower stressors (*p* < 0.001) compared to individuals who were dissatisfied with their choice. Similarly, OCSE-N and SNSS scores were statistically different among participants with respect to their perceived preparedness for nursing (*p* = 0.001 for both). Participants who reported they were prepared for nursing had significantly higher OCSE-N (*p* < 0.001) and lower stressors (*p* < 0.001) compared to individuals who reported feeling unprepared for nursing. Therefore, participants who reported greater satisfaction with their choice of pursing nursing and a greater perceived preparedness for the RN role had greater OCSE-N and lower stress. Consistent with the study aim, comparisons focused on intention-related and preparedness indicators measured during training; no further subgroup analyses were conducted.

## Discussion

The purpose of this study was to examine the relationship between occupational coping self-efficacy and perceived stressors as a nursing student with respect to nursing students’ professional intentions among undergraduate nursing students in Ontario, Canada. The findings revealed no statistically significant relationship between OCSE-N or SNSS scores and students’ plans to practice as a RN, pursue further education, or their preferred work setting and location. These results suggest that self-efficacy and stress levels may not directly influence Ontario nursing students’ professional intentions or postgraduate plans. This disconnect may indicate that more complex or mediating factors influence students’ long-term goals. For instance, students may perceive their current clinical unpreparedness or academic stress as temporary and inherent to professional growth, rather than a deterrent to becoming an RN. It is also possible that students’ passion for nursing, or a sense of ‘calling’, helps buffer the negative effects of stress and low self-efficacy. Moreover, encouragement from family, peers, and faculty may play a key role in maintaining students’ commitment to the profession, even under challenging conditions. These interpretations align with existing literature suggesting that a strong vocational commitment and social support promote program completion and long-term retention in nursing (Alharbi et al., 2019; Bauer & Kiger, 2017; Fowler & Norrie, 2009; Glogowska et al., 2007; Hamshire et al., 2013; Lott et al., 2018; Somers et al., 2019; Ten Hoeve et al., 2017; Tremblay et al., 2023; Ujváriné et al., 2011; Wray et al., 2014).

It is also important to consider the employment context of the sample when interpreting these findings. Nearly two-thirds of participants reported being employed during nursing education, and a substantial proportion were working in healthcare-related roles. Exposure to healthcare environments prior to graduation may foster adaptive coping strategies, normalize occupational stress, and strengthen vocational commitment, potentially attenuating the impact of stress and coping self-efficacy on longer-term professional intentions. Students who are already embedded within healthcare systems may perceive stressors as manageable or expected aspects of professional development rather than deterrents to future practice. This context may partially explain why OCSE-N and perceived stressors were not associated with plans to practice as a registered nurse or pursue further education in the present study. A notable finding in this study was that most students reported being satisfied with their choice to pursue nursing (77.1%), whereas only a small proportion were dissatisfied (10.6%). Participants with higher OCSE-N scores were more likely to report satisfaction with their career choice, while those with greater stress levels were more likely to express dissatisfaction. This introduces novel insights into the connection between occupational self-efficacy, perceived stress, and career satisfaction—areas not previously explored in depth. Prior research has demonstrated associations between self-efficacy, perceived clinical ability, and intent to pursue nursing (Alhowaymel et al., 2025). Extending this work, the current study demonstrates that higher occupational coping self-efficacy is associated with greater satisfaction with the decision to pursue nursing during training. The findings suggest that students’ confidence in managing occupational stressors may be meaningfully related to how positively they appraise their career choice while enrolled in nursing education.

Furthermore, this study expands the understanding of how stressors can affect nursing students’ satisfaction. Participants who were more satisfied with their career choice also reported fewer occupational stressors. This aligns with previous research demonstrating that excessive stress and unfavorable learning or working conditions can diminish students’ desire to continue in nursing (Lindberg et al., 2020; Williamson et al., 2013). In particular, Lindberg et al. (2020) highlighted how time pressure and challenging work environments can hinder nurses’ ability to deliver high-quality care, especially in inpatient settings, which may lead to dissatisfaction and avoidance of those roles.

Another key finding was that a substantial portion of the sample reported feeling either prepared (39.8%) or unprepared (38.1%) for the RN role. Participants who reported more prepared had higher OCSE-N scores and lower stress levels. These results reaffirm prior findings that stressors can undermine students’ sense of readiness for clinical practice (Ten Hoeve et al., 2017; Williamson et al., 2013). For example, the theory-practice gap, combined with high stress and inadequate working conditions, often leaves students feeling ill-equipped to transition into practice. This study confirms and extends those findings by demonstrating a clear link between occupational self-efficacy, stressors, and perceived preparedness.

While this study did not find a statistically significant relationship between OCSE-N and intent to practice as an RN, it did uncover a novel connection between OCSE-N and perceived readiness for professional practice. These results build on earlier work suggesting that students with greater perceived clinical ability are more likely to intend to enter the profession (Alhowaymel et al., 2025). Therefore, strategies aimed at strengthening students’ OCSE-N may contribute not only to increased preparedness for the RN role but also to long-term professional commitment. These findings offer meaningful implications for nursing education and warrant further research.

### Implications

The findings of this study have meaningful implications for nursing policy, practice, education, and research, particularly in addressing OCSE-N and student stressors. Enhancing students’ preparedness and satisfaction with nursing may positively influence their decision to remain in the profession after graduation. Specifically, targeted strategies to build clinical confidence and competence as students transition into practice are crucial. Gueroni et al. (2023) suggest that building self-efficacy is rooted in positive mental health, with the goal of instilling a belief in individuals that they have the ability to perform specific tasks. One effective approach to improve self-efficacy among nursing students could involve establishing formal mentorship programs, where trained mentors support students’ growth and confidence in their clinical skills. Furthermore, an integrative review by Gueroni et al. (2023) reported that among six clinical trials (both randomized and non-randomized) interventions emphasizing positive mental health (delivered over 8–12 weeks with psychoeducation and homework) strengthened participants’ self-efficacy, regardless of group size or delivery mode. The effectiveness of these interventions may stem from targeting positive mental health, a construct that promotes well-being through attributes such as personal satisfaction, autonomy, problem-solving, and interpersonal relationships. Incorporating these elements into clinical practice placements and nursing curricula may be particularly useful. Policy changes are needed to address systemic issues in the healthcare environment, such as short-staffing (Oshodi & Sookhoo, 2024), workplace violence (Cao et al., 2023), and bullying (Liao et al., 2025), which are factors that contribute significantly to student stress and dissatisfaction during clinical placements. Efforts to improve the workplace environment, particularly in direct patient care settings with limited resources or high stress, could make these roles more appealing and increase coping abilities and career satisfaction. By addressing these stressors, students may begin to view the transition into the RN role as achievable and rewarding. Optimizing staffing levels, creating supportive work environments, and ensuring fair recognition and compensation for nurses may foster a more positive perception of the profession. Such improvements have the potential to increase students’ occupational self-efficacy, reduce perceived stress, with potentially improved long-term retention in the nursing profession.

Implications for nursing education in Ontario also centre on developing OCSE-N and managing stress. Integrating self-efficacy-building activities and stress management techniques into undergraduate curricula may strengthen students’ resilience, clinical confidence, and satisfaction. Equally important is faculty development. Educators should be equipped with the tools and knowledge to foster students’ OCSE-N and understand the impact of stressors on student well-being. This would ensure that educators are better prepared to support students in managing stress, building resilience, and enhancing professional growth. These measures could improve students’ perceived clinical ability and reinforce their commitment to nursing.

Finally, the study has implications for future nursing research. A significant portion of participants had completed less than one year of their program (45.3%), and nearly one-third (31.9%) had not yet undertaken clinical placements. These factors likely influenced the lack of significant findings regarding occupational self-efficacy, stress, and professional intentions, as early-stage students may not yet have fully encountered the realities of the profession. Therefore, longitudinal research is recommended to examine how occupational self-efficacy, stress, satisfaction, and professional intentions evolve throughout the nursing program and during the transition to clinical practice. Overall, these findings underscore the importance of implementing strategies that reduce student stress and enhance self-efficacy which in turn can improve preparedness, satisfaction, and long-term commitment to nursing.

### Limitations

This study has some limitations. First, a large portion of the sample was drawn from a single institution (n = 149, 40.6%), potentially limiting the variability and representativeness of the Ontario baccalaureate nursing student population. The sample also underrepresented students enrolled in compressed-timeframe or bridging programs (n = 74, 5.4%) compared to those in four-year collaborative programs. Moreover, according to the Canadian Association of Schools of Nursing (2023), Ontario experienced a 30.8% decline in nursing program graduates in 2022 compared to the previous year (two years into the COVID-19 pandemic). As such, the current findings may not reflect the experiences or professional intentions of students who have left or failed to complete nursing programs. In addition, while it is possible that certain program structures may foster different perceptions of RN preparedness, our data did not include sufficient numbers from individual institutions to permit meaningful comparison. Another potential limitation is that institutional resources vary considerably across universities in Canada. Some well-endowed institutions may have greater access to simulation facilities, clinical placements, and academic supports compared to smaller or less resourced programs, which could influence students’ perceived preparedness for the RN role. These factors limit the generalizability of the results. A larger and more diverse sample might yield different or more statistically significant relationships among variables.

## Conclusion

In a large sample of Ontario baccalaureate nursing students, this study found no statistically significant relationship between occupational self-efficacy or student stress and professional intentions related to future practice or postgraduate education. However, higher self-efficacy and lower perceived stress were associated with increased satisfaction with the decision to pursue nursing and greater preparedness for the RN role. These findings emphasize the need to invest in strategies that enhance occupational self-efficacy and reduce educational stressors, with the ultimate goal of improving nursing students’ confidence, satisfaction, and long-term retention in the profession.
